# The Journal of the American Agricultural Association

**Published:** 1882-01

**Authors:** 


THE JOURNAL OF THE AMERICAN AGRICULTURAL ASSOCIATION. J. H. REAL, Editor. 127 Water street, New York.

"All national wealth depends on an enlightened agriculture." We welcome the above quarterly with more than usual pleasure. Its purpose is to disseminate the best attainable information on the subject of most importance to the farmer. It is a handsomely printed pamphlet of 260 pages, well illustrated and filled with articles at once clear, practical and profound. The essay on "The Railroad and the Farmer," by G. Edward Atkinson, is certainly an exceedingly valuable contribution. The railroad question has become of the utmost importance to the farmer of to-day, and Mr. Atkinson certainly presents the subject in a fair and favorable light for the consideration of the agricultural interest. Those who are fortunate enough to read Mr. Moulton's article on "Mountain Side Farm" will have new ideas of the degree of refinement and general excellence possible to farm life, as well as visions of the great importance of these elements to general agriculture as an art and as a science. Prof. Warder's article on "Agricultural Education in Bavaria" is full of thoughtful suggestions, as well as reflections on our own deficiencies in that department of instruction; "The Husbandry of the Ancients," by J. M. McBryde; "Agriculture in reland," by J. P. Sheldon; "Farmers and the Tariff," by Prof. Arthur L. Perry; "The Soil of the Blue-Grass Country," by W. E. Plumley; "Cotton Seed as Winter Feed," by J. H. Moore; "Preventable Losses," by Prof. I. P. Roberts; "The Need of Experiments," by Prof. J. P. Stelle; "Agriculture in the Tenth Census," by J. R. Dodge; "Seeds and Quality in Fruits," by Dr. E. L. Sturtevant; "Agricultural Instruction," by Byron D. Halsted; "Ripening of Wheat," by Prof. R. C. Kedzie; "The Railroad and the Farmer," by Edward Atkinson; and some comments on the last named article, by L. E. Chittenden. Some account is also given of the farm of the editor of the *Rural New-Yorker*. Our readers will perceive by this list of the articles in the volume the nature and character of its contents, several of which consist of papers read at the Cincinnati meeting of the Society for the Promotion of Agricultural Science. Space forbids a more extended notice of the contents of this volume. The contributors are all men of great intelligence, observation and practical experience. It is intended to issue it quarterly, at the modest price of $2 per year.

